# Office Visitors

**Published:** 1906-07

**Authors:** 


### OFFICE VISITORS.

He was an elderly colored man and a barber, with all the polite bearing and manners of the old time "befo de wah" negro slave. He had none of the crisp "yes and no", independant—I-am-as-much-of-a-man-as-you demeanor of the colored gentleman (?) of the present age. His every answer to our questioning was finished with the deferential "Sir." Not in humility, but in kindly feeling and respectful regard. "Doctor, I have cum to you in regards ter my teef, dey is gibbin me, wats leff ob dem, I've hed de mos ob dem pull out, er heap er trubble and I wants ter no if you tinks bess, fer me too hav'em tuck out an new false ones put een. I've cum ter you, Doctor, cause you an me is ob de ole set, an nose eech odder an I habs confydence een you, ter do me er good job. Bofe we, hab cum up foum de ole time, you nose my people and I nose yourn, an deres er feelin fer eech odder dat aint yuh munkst, dis yere new ginerashum er young wite peeple an black. Dey dont keer er copper fer eech odder, like we ole time folks dus. Look at my mout, Doctor, an tell me de bes ting ter do, an wat it gwine cos, I no youse gwinter be libral wid me." The examinotion made, determined us to extract few remaining loose teeth and put him in a set of artificial ones. Previous to the extraction he informed us that re "dident want nun of dat dere painless pizen dejected eenter my jaw. Why sur de odder day I wus er shavin er man and yered sicher racket up stairs ober my shop and dere wus es much es tree er fo mens er strugglin wid er man wat went ter dat dental parler doctor, who put sum er dat dere stuff een he jaw, an it like ter er kilt im. No Sur jess gib me er good drink ob wiskey an I'll stan it." When the set of teeth was finally adjusted to his satisfaction, he exclaimer. "Yes Docter dey is all rite and de gals tinks I'm a heap better lockin—yer no I'm er widdewer. Yes, sur, I stans sum chance now. Docter! would you mind faberin me wid er nudder drink ob yore wisky? Its mity fine—jes like de ole time stuff—not disyer spinsry licker." The draw stirred the cockles of his old heart in friendliness to us and he told us how kindly he felt towards the old time slave owners and said when "one ob den calls me er nigger, I likes it, it means fechun and kininess. Wen er wite man calls me Mister, I keeps my eye skinned on him." He went away clapping his hands together and laughing when we assured him that when we went to Heaven, if there were no old time "niggers" there we would turn about and walk out.

